# Mutations in *JAK/STAT* and *NOTCH1* Genes Are Enriched in Post-Transplant Lymphoproliferative Disorders

**DOI:** 10.3389/fonc.2021.790481

**Published:** 2022-01-17

**Authors:** Alexandra Butzmann, Kaushik Sridhar, Diwash Jangam, Hanbing Song, Amol Singh, Jyoti Kumar, Karen M. Chisholm, Benjamin Pinsky, Franklin Huang, Robert S. Ohgami

**Affiliations:** ^1^ Agilent Technologies, Santa Clara, CA, United States; ^2^ Department of Pathology, University of California, San Francisco, San Francisco, CA, United States; ^3^ Department of Pathology, Stanford University, Stanford, CA, United States; ^4^ Department of Laboratories, Seattle Children’s Hospital, Seattle, WA, United States

**Keywords:** PTLD, Epstein-Barr Virus (EBV), florid follicular hyperplasia, targeted sequencing, next generation (deep) sequencing (NGS), whole transcriptome sequencing

## Abstract

Post-transplant lymphoproliferative disorders (PTLD) are diseases occurring in immunocompromised patients after hematopoietic stem cell transplantation (HCT) or solid organ transplantation (SOT). Although PTLD occurs rarely, it may be associated with poor outcomes. In most cases, PTLD is driven by Epstein-Barr virus (EBV) infection. Few studies have investigated the mutational landscape and gene expression profile of PTLD. In our study, we performed targeted deep sequencing and RNA-sequencing (RNA-Seq) on 16 cases of florid follicular hyperplasia (FFH) type PTLD and 15 cases of other PTLD types that include: ten monomorphic (M-PTLD), three polymorphic (P-PTLD), and two classic Hodgkin lymphoma type PTLDs (CHL-PTLD). Our study identified recurrent mutations in *JAK3* in five of 15 PTLD cases and one of 16 FFH-PTLD cases, as well as 16 other genes that were mutated in M-PTLD, P-PTLD, CHL-PTLD and FFH-PTLD. Digital image analysis demonstrated significant differences in single cell area, major axis, and diameter when comparing cases of M-PTLD and P-PTLD to FFH-PTLD. No morphometric relationship was identified with regards to a specific genetic mutation. Our findings suggest that immune regulatory pathways play an essential role in PTLD, with the *JAK*/*STAT* pathway affected in many PTLDs.

## Introduction

Post-transplant lymphoproliferative disorders (PTLD) are abnormal lymphoid proliferations that develop in immunosuppressed patients after hematopoietic stem cell transplantation (HCT) or solid organ transplantation (SOT). The current 2017 revised World Health Organization (WHO) classification of hematopoietic neoplasms describes four subtypes of PTLD based on histological features: non-destructive, polymorphic, monomorphic and classic Hodgkin lymphoma type. These four subtypes reflect the phenotypic heterogeneity of PTLD (1). An important driver of PTLD pathogenesis is thought to be Epstein-Barr virus (EBV). About 70% of PTLD cases are reported to be associated with an EBV infection (2) and recent studies have investigated whether EBV positive and EBV negative PTLDs are distinct entities.

Other studies discuss the progression of PTLD from more benign subtypes, such as the early lesions (florid follicular hyperplasia, FFH) or polymorphic subtypes to more malignant subtypes of PTLD (i.e. monomorphic and classic Hodgkin lymphoma types) (3). Early lesions may be nearly indistinguishable from a reactive inflammatory response. However, next generation sequencing data may provide information about the molecular landscape and genetic profiles in PTLD, allowing for diagnostic subtyping, while also offering insight into the molecular pathogenesis and development of these diseases. To date, the published literature evaluating the genetic landscape of PTLD has been limited. These studies have shown distinct gene expression patterns and copy number aberrations in EBV positive PTLD as compared to EBV negative PTLD (3–5). Additional analyses have demonstrated distinct genetic mutations in monomorphic PTLDs with diffuse large B-cell lymphoma (DLBCL) (6, 7) or T-cell lymphoma phenotypes (8) compared to the corresponding lymphomas arising in immunocompetent patients.

Our study investigates the genetic DNA landscape and RNA gene expression profiles of ten cases of monomorphic, three cases of polymorphic and two cases of classic Hodgkin type PTLD. We compare these cases to the genetic landscape and gene expression profiles of 16 cases of early lesion FFH-PTLD using deep targeted DNA sequencing and RNA-profiling. We additionally evaluate nine cases of PTLD and FFH-PTLD by digital image analysis.

## Study Design

### Patient Cohort

For our study, we selected ten cases of monomorphic, three cases of polymorphic, and two cases of classic Hodgkin lymphoma type PTLD, and 16 cases of FFH-PTLD from the archives (2003-2018) of the Department of Pathology, University of California, San Francisco, Department of Pathology, Stanford University, and Department of Laboratories, Seattle Children’s Hospital. All cases and slides were reviewed and diagnoses were confirmed. Patient medical record charts, clinical and laboratory data as well as treatment data were re-reviewed. This study was approved by Institutional Review Boards at each site.

### Targeted Deep Sequencing

We extracted DNA from formalin fixed and paraffin embedded (FFPE) tissue using the DNA Storm Kit (Cell Data Science, CA, USA). For our DNA libraries, we developed a customized SureSelect^XT HS^ (Agilent Technologies, CA, USA) Heme Malignancies Evaluation and Infectious Detection panel (HeME-ID), which includes 354 genes that are important for lympho- or leukemogenesis in addition to 13 viruses and bacteria associated with hematolymphoid diseases (9, 10). 100 base-pair paired-end targeted deep sequencing was performed at an average coverage depth of 1500-fold on a high-throughput sequencing platform (HiSeq4000). We performed alignment using the Burrows-Wheeler Aligner – maximum exact matches (BWA-MEM) algorithm following the Genome Analysis Toolkit (GATK) best practices for alignment, single-nucleotide variant, and structural variant analysis. For variant calling, we used SureCall (version 4.1, Agilent Technologies). Analysis was run at a variant allele frequency (VAF) of 2%, which was justified by the high read depth and the usage of molecular barcodes in the Sureselect^XT HS^ kit. In order to call a mutation, a 20x read coverage per base and a minimum coverage in forward and reverse direction were also required. We applied the same filters for small insertion and deletions (indel) analysis. Annotation of variants was performed using SureCall and wAnnovar (11). For further curation we applied filters of a maximum VAF of 40% and a minimum Combined Annotation Dependent Depletion (CADD) score of 20. Synonymous mutations and mutations outside of exons where excluded. We also used SureCall for analysis of structural variants. For further downstream analysis, such as enrichment or depletion analysis in the promoter region, promoter flanking regions and transcription factor binding sites (CTCF), as well as the analysis of the mutational signature, we used the MutationalPatterns (12) Package from R. Pathway Analysis along with EnrichR (13), Gene Set Enrichment Analysis (GSEA) (14, 15) and ConsensusPathDB-human (CPDB) (16). Evaluation of microorganisms was performed using the subtraction method as described for shotgun metagenomic sequencing (17, 18). For viruses, we interpreted the results based on percent coverage of the targeted regions and average depth. Based on our previous studies, we classified samples as: negative, equivocal, and positive. In order to be interpreted as equivocal, all three viral targeted regions must have a coverage of at least 10% up to 75% and the average read depth must be at least 1. For a sample to be classified as positive, all three targeted regions of a sample required a coverage minimum of 75% and an average read depth of at least 9.

### RNA-Sequencing and Data Analysis

RNA was extracted from FFPE tissue blocks with the RNA Storm FFPE DNA Extraction kit (Cell Data Science). The quality and quantity of extracted nucleic acids was assessed by Qubit analysis and 2100 Bioanalyzer (Agilent). We used 200ng of RNA to prepare our RNA library with the SureSelect^XT^ RNA Direct kit (with SureSelect Exome V6 + UTR Capture Library) for strand-specific sequencing libraries (Agilent Technologies). We performed 150 base-pair paired-end whole transcriptome sequencing on a high-throughput sequencing platform from Illumina (HiSeq4000) for an average coverage of 300 million reads per sample. For downstream processing of our output files, we used Hisat2 (version 2.1.0) for alignment and HT-Seq (version 0.11.1) for generation of the count files. Gene expression analysis was performed in RStudio (version 3.5.3). Primary analysis of expression data was performed with ClustVis (19). We used the DeSeq2 package for differential gene expression analysis (20). For fusion analysis, we used STAR-Fusion (version 0.1.1) (21). The immunologic environment was analyzed by CIBERSORT (22).

### Computational Digital Image Analysis

We performed digital imaging analysis of representative cases from both the M-PTLD, P-PTLD (n=9) and FFH groups (n=9). MATLAB (v2019B) was used to analyze the RGB images acquired from an Aperio AT2 scanner at an optical magnification of 40X. K-means clustering was used to differentiate the different cellular components and segment the images (23). The image processing toolbox from MATLAB was used to extract eight parameters from the image datasets, which include: area, circularity, major axis, minor axis, eccentricity, equivalent diameter, solidity, and perimeter. Welch’s t-test was used to perform a two-tailed test on these parameters.

### Statistical Analysis

All statistical analysis was done in RStudio (R version 3.6.0 and RStudio Version 1.2.1335). ﻿Student’s t-test was performed to evaluate differences between datasets. A p-value < 0.05 was considered statistically significant.

## Results

### Patient Cohort

Our study included 15 patients diagnosed with advanced PTLD and 16 patients with FFH. The cases of advanced PTLD included ten cases of monomorphic, three cases of polymorphic, and two cases of classic Hodgkin lymphoma type PTLD. The diagnoses for all cases were reconfirmed and classified by R.S.O., B.P., J.K. and K.C. and A.B. based on the 2017 revised WHO classification of lymphoid neoplasms. Our patient cohort consisted of 12 females and 19 males. The average age of the non-FFH PTLD group was 40 years old (range 6 to 67 years) and the average age in the FFH group, 8 years old (range 2 to 20 years). Twelve patients were positive for EBV by *in-situ* hybridization (ISH) in the non-FFH PTLD group, and six patients were positive for EBV by ISH in the FFH group. The clinical characteristics of the non-FFH PTLD and FFH groups are provided in **Tables 1**, **2**, respectively.

**Table 1:** Clinical and histological characteristics of the PTLD patients included in study.

Case	PTLD subtype	EBV status (ISH)	EBV Status (NGS)	Sex	Age	HCT/SOT Indication	Treatment
4	CHL	+	+	F	48	Relapsed HL	Rituximab
6	Monomorphic (DLBCL)	+	+	M	66	BPDCN	Rituximab
28	Polymorphic	+	+	M	24	Relapsed HL	NA
30	T-cell	+	–	M	67	High risk CLL	Nilotinib
32	Monomorphic (DLBCL)	Scattered +	–	M	18	CM	POG 9219
27	Monomorphic (DLBCL)	+	+	M	52	Pre-B-ALL	Rituximab
8	Monomorphic (DLBCL)	–	–	M	56	Cirrhosis	R-CHOP
25	CHL	Scattered +	+	M	24	CM	Stanford V chemotherapy followed by involved field radiotherapy 30 Gy in 20 fractions directed at the pre-chemotherapy disease
13	Polymorphic	+	+	M	22	Relapsed HL	Rituximab
11	Monomorphic (DLBCL)	+	+	F	58	PCKD	R-CHOP
17	Monomorphic (DLBCL)	+	+	F	44	Cirrhosis	Rituximab
29			+				
21	Polymorphic	+	equivocal	M	23	Aplastic Anemia	NA
24	Monomorphic (DLBCL)	–	–	F	40	ESRD	R-CHOP
23	T-cell	+	+	M	6	ESRD	CHOP + high-dose cytarabine with asparaginase
20	Monomorphic (DLBCL)	–	–	F	54	Cirrhosis	Rituximab

PTLD, post-transplant lymphoproliferative disorder; pre-B-ALL, pre-B-cell acute lymphoblastic leukemia; BPDCN, blastic plasmacytoid dendritic cell neoplasm; CHL, classic Hodgkin lymphoma; CLL, chronic lymphoblastic leukemia; CM, cardiomyopathy; HL, Hodgkin lymphoma; DLBCL, diffuse large B-cell lymphoma; ESRD, end-stage renal disease; PCKD, polycystic kidney disease; R-CHOP, rituximab-cyclophosphamide, doxorubicin, vincristine, prednisone. N/A, not applicable.

**Table 2:** Clinical and histological characteristics of the patients with florid follicular hyperplasia included in study.

Case	PTLD subtype	EBV status (ISH)	EBV Status (NGS)	Sex	Age	HCT/SOT Indication
15	FFH	–	–	M	20	ESRD
14	FFH	–	+	M	5	CM
26	FFH	–	–	M	8	ESRD
22	FFH	–	–	F	4	ESRD
7	FFH	–	–	F	2	Cirrhosis
16	FFH	–	–	F	4	PCKD
3	FFH	+	+	F	6	NEC
19	FFH	+	+	M	4	Biphenotypic AML
2	FFH	+	+	M	2	Cirrhosis
10	FFH	Scattered +	+	F	13	CM
18	FFH	–	–	F	20	ESRD
12	FFH	+	+	M	9	Cirrhosis
5	FFH	–	–	M	2	ESRD
31	FFH	–	equivocal	M	14	ESRD
9	FFH	+	+	M	18	CM
1	FFH	–	+	F	3	CM

PTLD, post-transplant lymphoproliferative disorder; FFH, florid follicular hyperplasia; AML, acute myeloid leukemia; CM, cardiomyopathy; ESRD, end-stage renal disease; NEC, necrotizing enterocolitis; PCKD, polycystic kidney disease.

### Mutational Analysis Reveals Recurrent JAK3 Mutations in Monomorphic, Polymorphic, and Classic Hodgkin Lymphoma PTLD Cases

We performed targeted deep sequencing to gain insight into the mutational landscape of M-PTLD, P-PTLD, and CHL-PTLD in comparison to FFH. The average number of mutations after curation in our M-PTLD, P-PTLD and CHL-PTLD cases was 10.8 and in the FFH cases was 2.8 (with only 10 of 16 FFH cases carrying mutations). The mutations found in the non-FFH PTLD cases were significantly more deleterious than the mutations found in FFH based on the CADD score (average CADD score 28.52 vs. average CADD score 18, p-value 0.01). Among the histological subtypes, the polymorphic subtype had the smallest number of mutations, and the somatic mutations were less damaging. In comparison, other histological subtypes, including monomorphic (DLBCL histology), and classical Hodgkin lymphoma, had a higher number of mutations with more damaging somatic mutations (**Table 3**). Variants for the PTLD cases had a VAF ranging from 2 to 37% with an average of 4.7%, whereas the VAF for the alterations detected in the FFH cases ranged from 2 to 6% with an average of 2.8%.

**Table 3:** Summary of average number of mutations identified and average CADD score for PTLD subtypes and EBV infection status.

	CHL	T-cell	Monomorphic (DLBCL)	polymorphic	PTLD EBV+	PTLD EBV-	FFH EBV+	FFH EBV-
Average number of mutations	31.5	9	8.4	5.3	10.73	10.8	3	2.57
Average CADD score	30.34	28.86	28.62	25.51	27.88	29.16	15.59	20.42

CHL, classic Hodgkin lymphoma; DLBCL, diffuse large B-cell lymphoma; PTLD, post-transplant lymphoproliferative disorder; FFH, florid follicular hyperplasia; EBV, Epstein-Barr virus.

**Figure 1:**
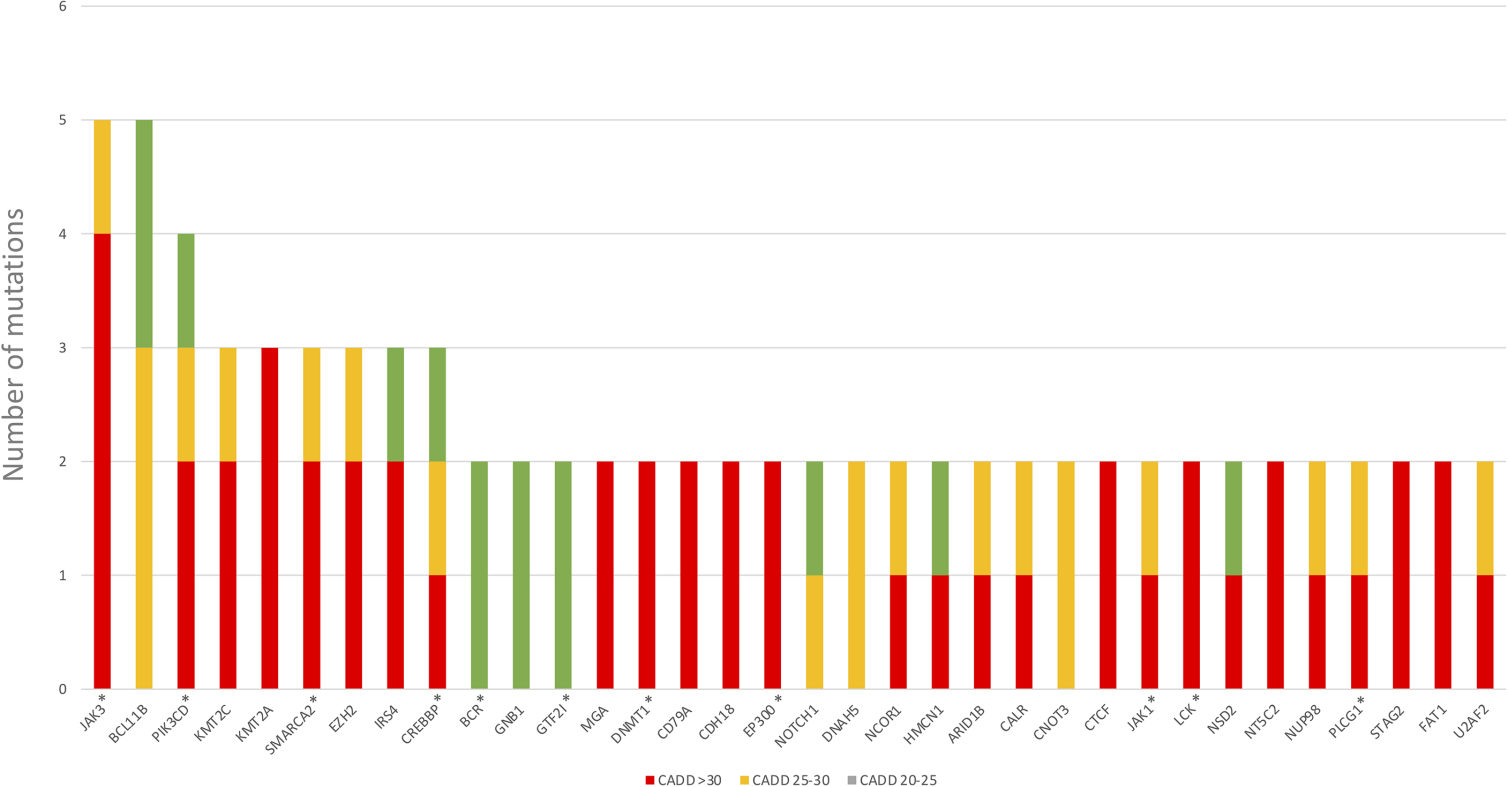
Genes mutated in more than one PTLD sample. *Genes affecting the *JAK/STAT* pathway.

**Figure 2:**
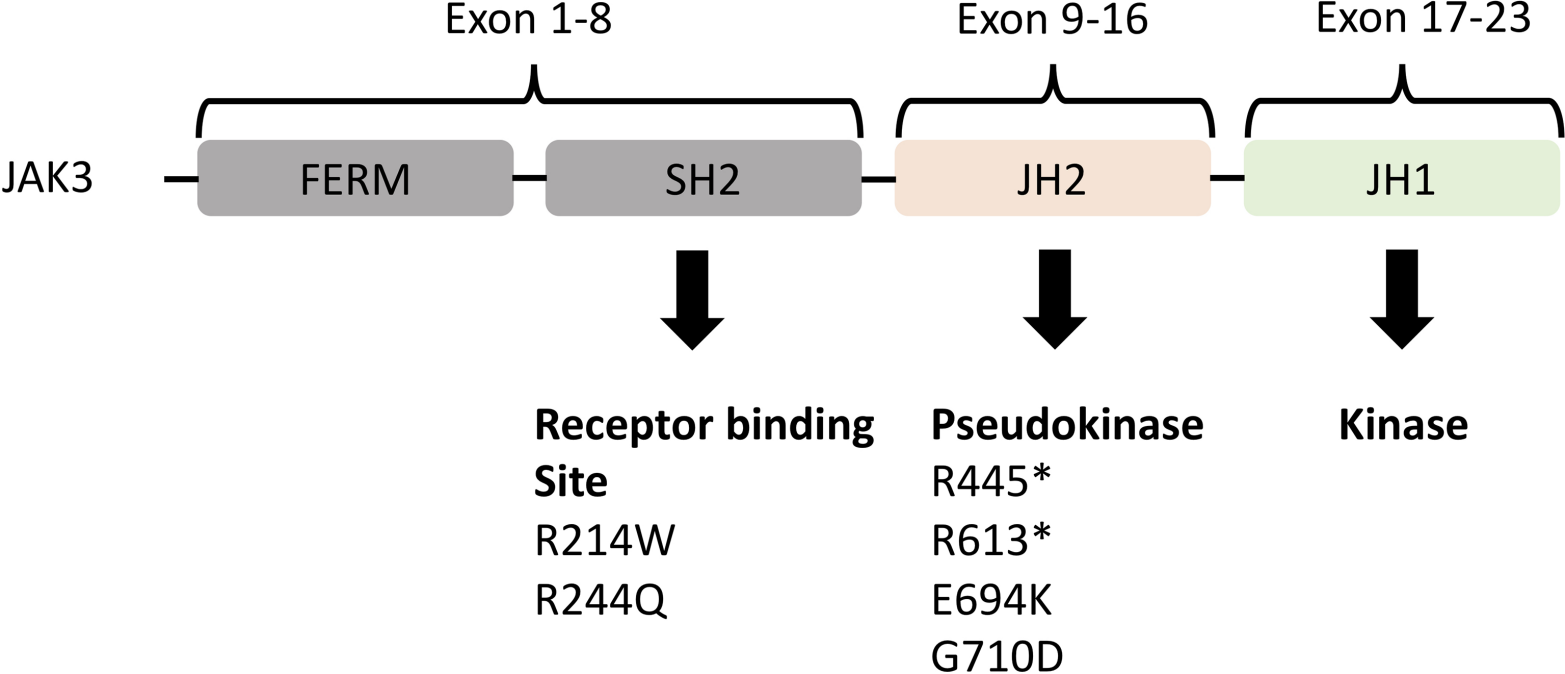
Representation of the JAK3 domains and their encoding exons (24, 25) with JH2 (pseudokinase domain) and JH1 (kinase domain) being the most important. Below the domains are the somatic mutations identified in both M-PTLD, P-PTLD, CHL-PTLD and FFH-PTLD patients. *nonsense mutation.

**Figure 3:**
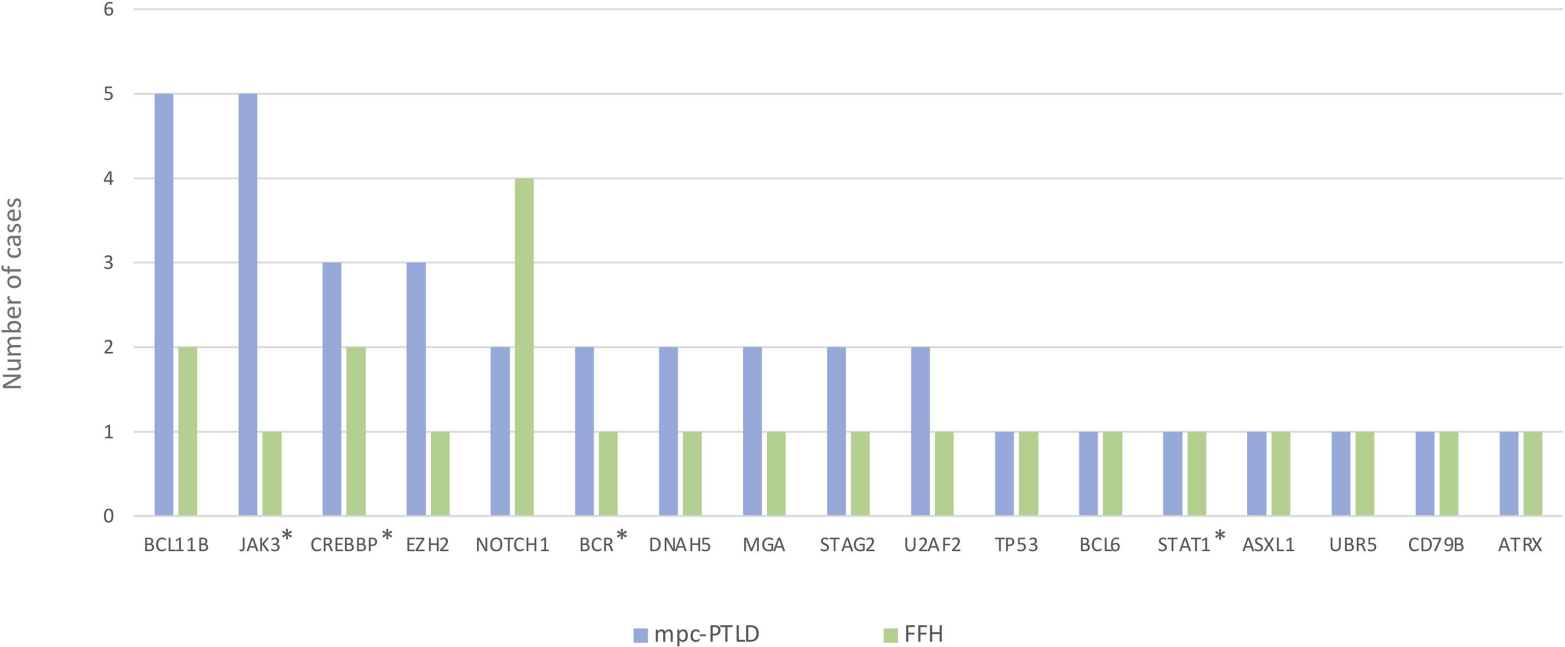
Genes mutated in the mpc-PTLD and FFH-PTLD groups. *Genes affecting the *JAK/STAT* pathway.

We also discovered *JAK3* and *BCL11B* mutations in three M-PTLD cases, two CHL-PTLD cases, and *PIK3CD* mutations in four M-PTLD cases. Other genes found to be mutated in more than one case are illustrated in **Figure 1**. Of the genes mutated in greater than three patients, *JAK3* mutations were classified as deleterious based on CADD scores, whereas *BCL11B* mutations had lower CADD scores. *JAK3* mutations were seen in the SH2 and JH2 domains (**Figure 2**). Pathway analysis of the recurrently mutated genes revealed that those genes are key in the *JAK/STAT* pathway and cytokine signaling pathways. Genes affecting the *JAK/STAT* pathway were marked in **Figures 1**, **3**. These genes were also involved in IL-2, IL-3, IL-5 and IL-7 and GM-CSF signaling pathways and signaling events mediated by T-cell Protein Tyrosine Phosphatase (TCPTP).

### Overlapping Gene Mutations in M-PTLD, P-PTLD, CHL-PTLD and FFH From Post-Transplant Patients

**Figure 4:**
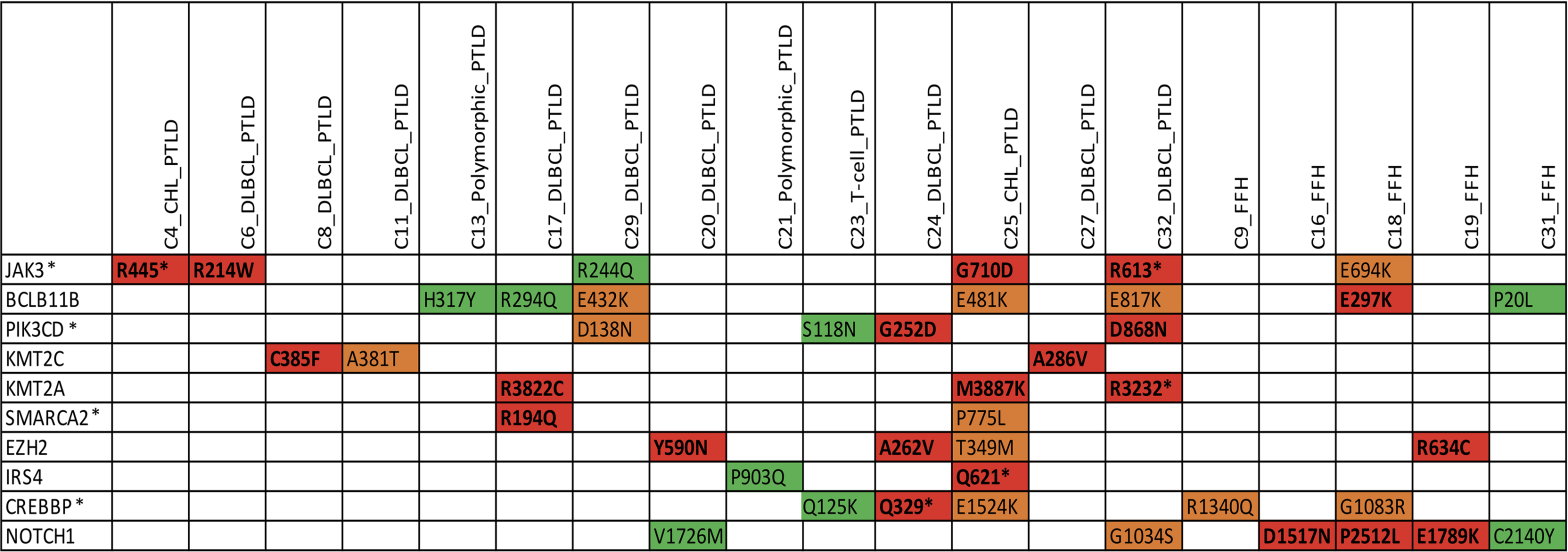
Overview of somatic mutations in recurrently mutated genes in the mpc-PTLD and FFH-PTLD groups. Somatic mutations with a CADD score of >30 are shown in red, CADD score of 25-30 in orange, and CADD score of 20-25 indicated in green. *Genes affecting the *JAK/STAT* pathway.

We identified somatic mutations in 17 overlapping genes between the M-PTLD, P-PTLD, CHL-PTLD and FFH groups (**Figure 3**). The genes recurrently mutated in both groups include *NOTCH1* (four patients), *CREBBP*, and *BCL11B*. All but one mutation in the *NOTCH1* gene were deleterious. The pathogenicity of the somatic mutations involving genes mutated in more than three patients is shown in **Figure 4**.

### More Mutations Than Expected in Non-Coding Gene Regions in M-PTLD, P-PTLD, CHL-PTLD and FFH

**Figure 5:**
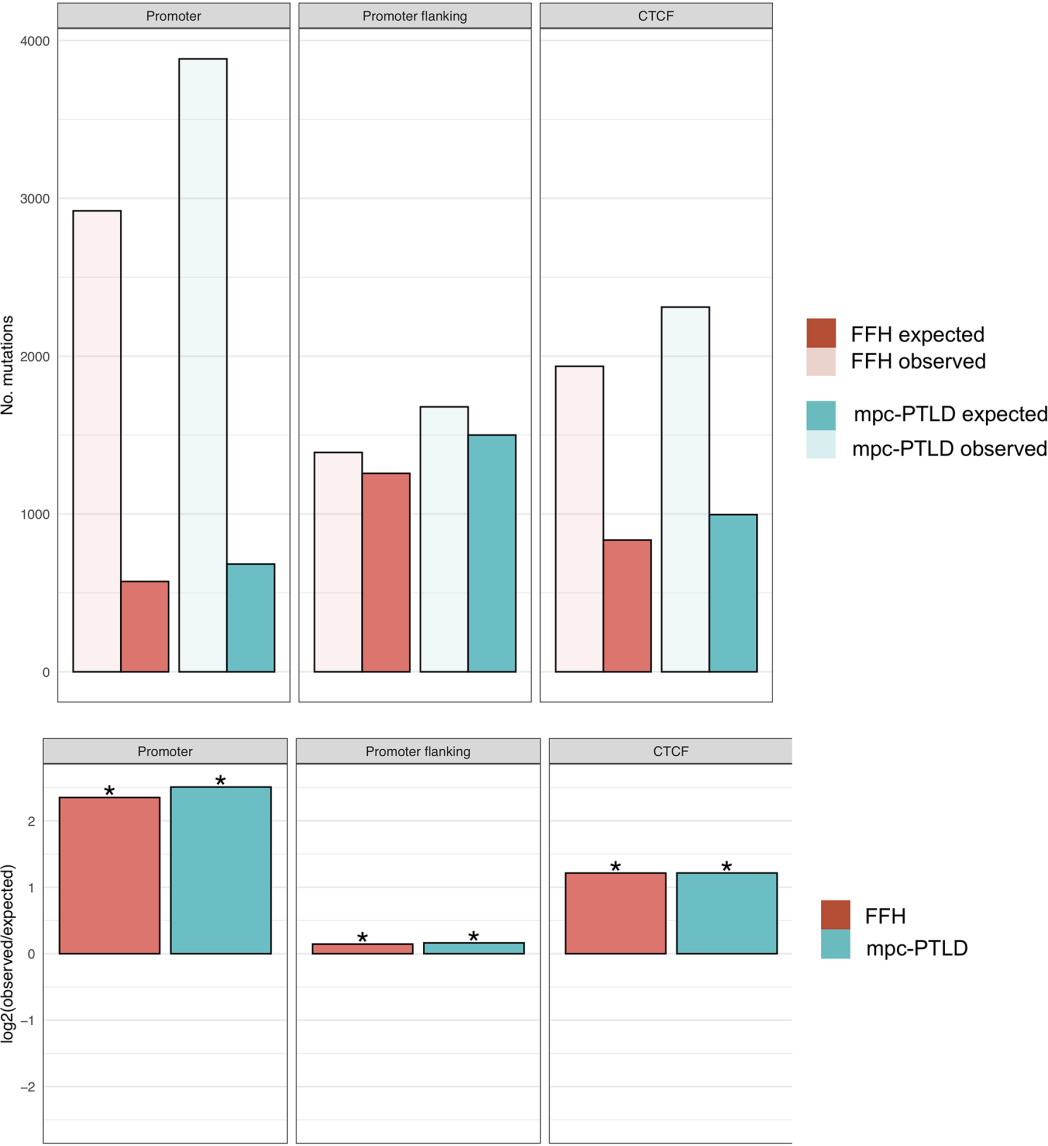
Enrichment analysis for promoter region, promoter flanking regions, and transcription binding sites (CTCF). The top part of the figure shows the observed versus expected number of mutations. In the bottom part of the figure, the mutational burden within the same non-coding gene regions for both groups are demonstrated as the log2 ratio of the number of observed and expected point mutations indicating the effect size of the enrichment or depletion within each region. (*) = statistical significance (p<0.05; two‐sided binomial test).

In order to better understand the overall mutational burden and the distribution of somatic mutations within our samples and groups, we also evaluated the non-coding gene regions, such as promoter, promoter flanking and transcription factor binding sites (CTCF). Here we noted an overall higher number of mutations in M-PTLD, P-PTLD, and the CHL-PTLDs versus FFH, in the promoter regions compared to three non-coding regions (**Figure 5**).

### The Mutational Landscape and Gene Expression Profile in M-PTLD, P-PTLD, and CHL-PTLD Is Unrelated to EBV Infection

We performed mutational analysis and evaluated the EBV infection status for M-PTLD, P-PTLD, CHL-PTLD and FFH cases. EBV infection status was determined by ISH and NGS using our targeted HeME-ID panel. By NGS, ten non-FFH PTLD cases and eight FFH cases were positive for EBV infection in the analyzed tissue (**Table 2**). There was no difference in the number of mutations identified between the groups of EBV infected and non-infected patients (**Table 3**). The somatic mutations found in infected patients had similar CADD scores of pathogenicity, p-value 0.69 (**Table 3**). Discrepancies between the ISH and NGS findings can be explained by two primary factors. The layer of tissue obtained from sectioning the block is variable and may account for the differences between the ISH and NGS studies (i.e. sampling variability). Secondly, interpretation of ISH is subjective, whereas NGS uses predefined parameters with less variation.

**Figure 6:**
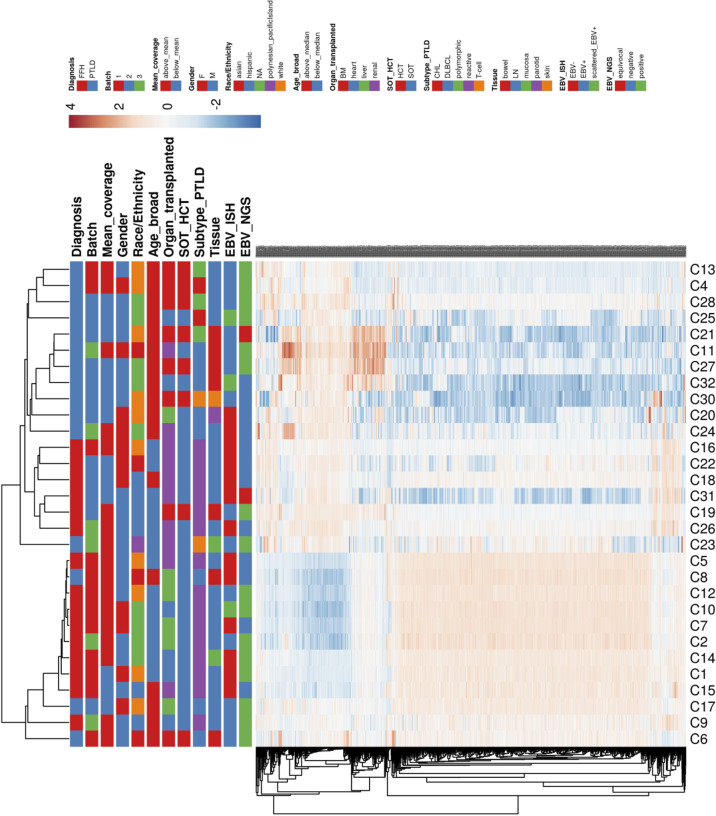
Heatmap hierarchically clustered by Euclidean distance of gene expression for all of the investigated groups (see legend).

Hierarchical clustering of the gene expression profiles shows primary clustering based on the diagnosis of mpc-PTLD and FFH. We also looked for a distinct transcriptional profile based on EBV infection status and noted that EBV negative cases had a tendency to cluster together (**Figure 6**). However, there was no clear separation between EBV positive and negative cases in the gene expression profiles, which was further confirmed using principal component analysis (PCA) (**Supplementary Figure 1**). The different expression patterns did not appear to be associated with any of the other factors we investigated, including batch, mean coverage, gender, race/ethnicity, age, organ transplanted, SOT/HCT, PTLD subtype, or tissue type.

### Genes Involved in Regulatory or Innate and Adaptive Immune System Are Upregulated in mpc-PTLD

We performed RNA-sequencing to understand the gene expression profiles of mpc-PTLD compared to FFH. In our exploratory analysis, we saw that mpc-PTLD patients have a different gene expression profile as compared to FFH patients with a subset of mpc-PTLD cases falling in between (cases 6, 8, 17 and 23), as demonstrated in **Figure 6**. We looked at the differentially expressed genes by gene set enrichment analysis and found that genes involved in regulatory or innate and adaptive immune system are overexpressed in mpc-PTLD as compared to FFH.

### mpc-PTLD Cases Have More T-Cell Involvement and Fewer B-Cell Involvement Than FFH Cases

**Figure 7:**
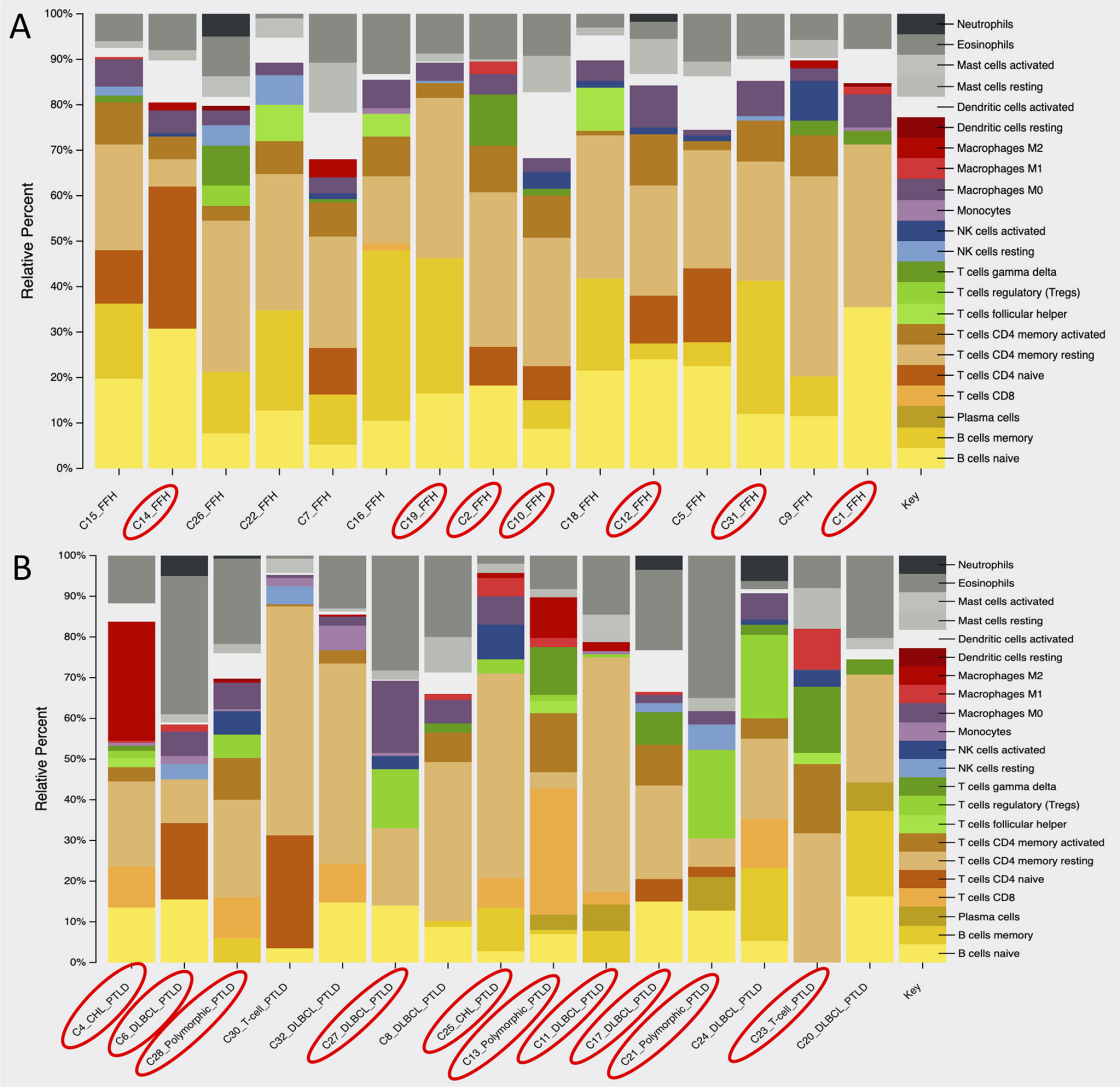
Analysis of the immune cell composition within **(A)** the FFH-PTLD cases and **(B)** the monomorphic, polymorphic, and classic Hodgkin (mpc)-PTLD cases based on gene expression profile. EBV positive cases are circled in red.

Using the gene expression profile of our samples, we looked at the immune cell composition within the mpc-PTLD and FFH group. As shown in **Figure 7**, there is a greater B-cell component (naïve and memory) within the FFH group, whereas the mpc-PTLD group has a stronger overall CD8 and CD4 T-cell component. In general, the mpc-PTLD cases have a more heterogenous immune cell infiltration with more T-follicular helper cells and a greater mast cell component in some of the cases. The mpc-PTLD cases diagnosed with a T-cell subtype show strong signal for T-cells, while lacking a significant B-cell component.

### Digital Imaging Analysis Demonstrates That mpc-PTLD Cells Are Larger Than FFH Cells

**Figure 8:**
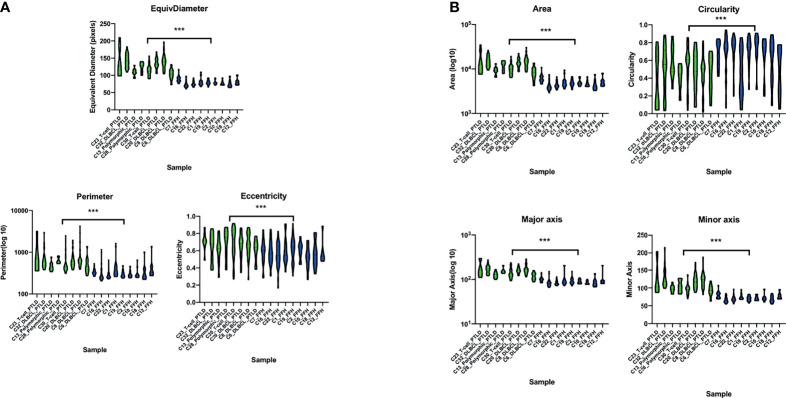
Violin plot of the digital imaging analysis of M-PTLD, P-PTLD, CHL-PTLD, and FFH-PTLD cases. **(A)** Violin plots of cellular parameters of equivalent diameter, perimeter and eccentricity. **(B)** Violin plots of cellular parameters of area, circularity, major axis, and minor axis. Displayed are nine representative samples of each group. Green represents M-PTLD and P-PTLD cases, blue indicates FFH-PTLD cases. (***) = statistical significance (p<0.0001).

We performed digital imaging analysis of nine representative cases of both the mpc-PTLD and FFH groups. In our analysis, we found that mpc-PTLD cells have a significant larger area, diameter, and major axis (**Figure 8**) (p<0.0001). There was no significant difference in the circularity, minor axis, eccentricity, or perimeter. We were particularly interested in cases that seems to have a molecular overlap. Cases 6, 8 and 23 were analyzed by digital imaging and their transcriptional profile appears more similar to the transcriptional profile of FFH (**Figure 6**). We were unable to identify differentiating features among cases 6, 8, and 23 as well as the remaining cases of the mpc-PTLD group.

## Discussion

In our study, we investigated and compared the molecular landscape of 15 cases of advanced PTLD and 16 cases of FFH. Here we performed a high-throughput molecular comparison and conducted digital imaging analysis of mpc-PTLD and FFH cases.

Our mutational analysis showed a higher number of mutations for the mpc-PTLD cases with somatic mutations that were more deleterious than those in the FFH cases. We identified somatic mutations in all of the mpc-PTLD cases but only in 10 of 16 FFH cases. These findings were further confirmed by evaluating the mutational burden of the specific non-coding regions: promoter region, promoter flanking and transcription factor binding sites (CTCF) region. Here we again saw a higher number of mutations within the mpc-PTLD group. Interestingly, we observed more promoter region mutations than expected for both mpc-PTLD and FFH. Overall, we found both groups to be very heterogenic with respect to the number of mutations identified and the mutational landscapes.

Previous studies reported on PTLD arising from early lesions, such as FFH (26, 27). Our analysis further corroborates these findings and we identified 17 overlapping genes mutated in both the PTLD and FFH groups. The majority of these genes were mutated in more than one mpc-PTLD case. Among the overlapping genes detected in both mpc-PTLD and FFH, *JAK3*, *BCL11B*, and *PIK3CD* were recurrently mutated in mpc-PTLD in four or more cases. Five of the six detected mutations in *JAK3* were deleterious, all with very low VAF (3-10%). Although we used FFPE tissue for targeted deep sequencing, we were able to detect very low VAF based on a high read depth and the use of molecular barcodes in our library chemistry. *JAK3* is a cytokine receptor and plays a critical role in the *JAK/STAT* pathway. The *JAK/STAT* pathway plays an important role in the regulation of cell proliferation and immune system response, especially by involvement of cytokine and interleukin signaling (28). *JAK3* mutations have been reported in mainly T-cell neoplasms (29,) (30), immunodeficiency syndromes (31, 32), and B-cell neoplasms (33). The mutations found in our PTLD and FFH cases fall into the SH2 and JH2 domains of the *JAK3* gene. The JH2 pseudokinase domain is the most commonly affected domain within all the *JAK* mutated genes involved in hematolymphoid diseases, with most mutations functioning as activating mutations (34). The pseudokinase domain suppresses the directly adjacent kinase domain JH1 (24). The SH2 domain is located in the receptor binding domain where kinase activity is initiated and the JAK receptor specificity is determined (35,) (36). Given that current drugs, such as tofacitinib and peficitinib, target the *JAK3* signaling pathway, our findings support the potential role of JAK3-targeted therapy to improve treatment options for PTLD (37–39). However, more cases are necessary to confirm the recurrence of *JAK3* mutations in PTLD. Further, functional studies are necessary to determine if the discovered mutations are activating or inactivating mutations.

Within the FFH group, *NOTCH1* was recurrently mutated. *NOTCH1* mutations often occur in T-cell acute lymphoblastic leukemia/lymphoma (T-ALL) (40) and multiple studies have demonstrated the importance of NOTCH1 signaling for the induction of lymphomagenesis (41–43). A recent study by Kimura et al. suggested that activating *NOTCH1* mutations play a role in the genetic evolution of pediatric T-ALL (44). The mutation detected in our cases are mostly deleterious with two of five cases involving the PEST domain of *NOTCH1* (exon 34). This PEST domain is one of the two most frequently affected domains, often a result of activating mutations (41). Our findings of recurrent *NOTCH1* and overlapping mutational landscapes support the published literature suggesting possible progression of FFH to monomorphic, polymorphic, and classic Hodgkin lymphoma type PTLD. Our FFH study group was younger than the mpc-PTLD group which may lead to differences in outcome and genetic landscape between the two study groups (45).

We performed RNA-sequencing to compare the gene expression profiles of our mpc-PTLD cases with FFH. We found that the FFH cases show a distinct gene expression profile as compared to the mpc-PTLD cases. However, some mpc-PTLD cases exhibited a hybrid expression profile relative to the FFH cases, indicative of a possible relationship between the two groups. Differential gene expression analysis showed an overexpression of genes involved in the innate and adaptive immune system as well as complement activating genes. These results underline our mutational findings of recurrently mutated *JAK3* and *NOTCH1*, both of which are important in immune system regulation.

Through our targeted deep sequencing analysis, we found that the variants for the PTLD cases had a greater average VAF as compared to the VAF for the alterations detected in the FFH cases. These findings suggest that the lower average VAF in cases of FFH may represent gene expression of a benign or reactive background environment whereas the higher average VAF in the mpc-PTLD group may indicate a gene expression pattern of neoplastic cells. Although the majority of the mpc-PTLD group consisted of monomorphic PTLDs, we also compared the number of mutations of the other histological PTLD subtypes. We found that the polymorphic subtype had the smallest number of mutations with less damaging somatic mutations. In concordance with the current literature, we noted that polymorphic PTLD may be a precursor lesion to the other subtypes and we believe our data supports these findings (3). However, given the small sample size within this group, interpretation of these results is limited. In addition, as mentioned before the FFH study group was younger than the mpc-PTLD study group which may cause a difference in genetic landscapes.

Using our HeME-ID panel that targets 13 viruses and bacteria, we evaluated the EBV infection status of all 31 patients. EBV infection was seen in ten out of 15 mpc-PTLD tissues and nine of 16 FFH tissues. Through mutational analysis, we identified a similar number of mutations for EBV positive and EBV negative cases. The pathogenicity of the mutations found in the EBV positive cases was lower than in the EBV negative cases. Our results support the findings of Menter et al., who found that EBV positive monomorphic PTLD cases had a less pathogenic mutational landscape as compared to EBV negative cases and suggested that EBV induces lymphomagenesis through its oncogenic properties, behaving as a substitute for deleterious mutations (7). When comparing the gene expression profiles, two studies found differences in the gene expression pattern of EBV positive and EBV negative PTLD cases (4, 5), whereas another study did not detect any differentially expressed genes (27). We also noted that the EBV negative cases clustered in between the positive cases in the hierarchical clustering analysis. However, no genes were significantly differentially expressed based on EBV infection status.

To study the immunologic environment of our mpc-PTLD and FFH cases, we looked at the immune cell composition based on the gene expression profiles of the two groups. We found that B-cells were the predominant cell type within the FFH group, whereas the mpc-PTLD group consisted of a large proportion of CD4 and CD8 cells, T-follicular helper cells, and mast cells. Overall, the immune cell composition of the mpc-PTLD cases was more heterogenic and was also unrelated to the EBV infection status, as noted in **Figure 7**. This finding is important to note since the dominating CD4/CD8 T-cell component within the mpc-PTLD group has been associated with EBV infection status due to the naturally occurring T-cell immune response seen in EBV positive immunocompetent patients (8, 46). Based on our molecular findings, we hypothesize that a change of the immune cell composition in PTLD may be due to the dysregulated immune response. *JAK/STAT* and *NOTCH1* pathway defects are often seen in T-cell malignancies (28) and may explain the distinction in B-cell and T-cell composition between the mpc-PTLD and FFH groups. Moreover, *JAK/STAT* is a major regulator of cytokine pathways and dysregulation may also lead to increased mast cells in the immunologic environment. Overall, our immune cell composition findings support our molecular findings. Future studies are also warranted to determine whether the cell of origin in PTLD is recipient- or donor-derived as this may provide further insight into the immune-mediated pathways involved in PTLD.

To our knowledge, this is the first study to perform digital analysis on whole slide images of PTLD samples. The histologic presentation of PTLD is very heterogenic, and it can be difficult to distinguish from other benign or malignant processes of the lymph node (47). Thus, we utilized digital analysis of whole slide images to determine if the cells from monomorphic, polymorphic, or classical Hodgkin type PTLD can be distinguished from FFH cells. mpc-PTLD cells were significantly larger in area, diameter, and major axis as compared to the FFH lymphocytes (p<0.0001). These results were expected as mpc-PTLD lymphocytes can be enlarged and can be confused with other entities, such as a plasma cell neoplasm (48). Surprisingly, mpc-PTLD and FFH lymphocytes showed no significant differences in circularity, eccentricity, perimeter, and minor axis since we anticipated mpc-PTLD and FFH lymphocytes to be more distinguishable. Our findings support the molecular data and show similarities between m-PTLD, p-PTLD, CHL-PTLD and FFH lesions. Of note, our imaging analysis was limited by the number of cases and disease entities in this study. Greater sample sizes are needed to perform an in-depth analysis of the morphological hallmarks of PTLD pathology.

## Conclusion

Our study is the first comprehensive analysis evaluating the molecular landscapes of monomorphic, polymorphic, or classic Hodgkin type PTLD and FFH. Limitations of this study are the small sample size as well as the age difference between mpc-PTLD and FFH group. However, our findings contribute to a better understanding of the pathogenesis of PTLD and will help guide future functional studies for these disease processes.

## Data Availability Statement

The data presented in the study are deposited in the Genbank repository, accession numbers SAMN24663821-SAMN24663850 or available upon request to the corresponding author.

## Ethics Statement

The studies involving human participants were reviewed and approved by the UCSF Institutional review board. Written informed consent from the participants’ legal guardian/next of kin was not required to participate in this study in accordance with the national legislation and the institutional requirements.

## Author Contributions

RO conceived of and designed the study, analyzed data, and edited the manuscript. BP conceived of and designed the study, and edited the manuscript. AB designed the study, performed research, designed experiments, analyzed data, and wrote the manuscript. KS, DJ, HS, AS, JK, KC, and FH analyzed data and edited the manuscript. All authors contributed to the article and approved the submitted version.

## Conflict of Interest

The authors declare that the research was conducted in the absence of any commercial or financial relationships that could be construed as a potential conflict of interest.

## Publisher’s Note

All claims expressed in this article are solely those of the authors and do not necessarily represent those of their affiliated organizations, or those of the publisher, the editors and the reviewers. Any product that may be evaluated in this article, or claim that may be made by its manufacturer, is not guaranteed or endorsed by the publisher.

## Supplementary Material

Supplementary Figure 1 PCA plot of the transcriptional profile of **(A)** EBV positive and EBV negative monomorphic, polymorphic, and classic Hodgkin (mpc)-PTLD cases and **(B)** EBV positive and EBV negative FFH-PTLD samples. EBV status was determined by NGS.
