# Supplementary figures and images for: Mutations in JAK/STAT and NOTCH1 Genes Are Enriched in Post-Transplant Lymphoproliferative Disorders
Source: Front Oncol. 2022 Jan 17;11:790481. doi: 10.3389/fonc.2021.790481 (PMC8801788; doi:10.3389/fonc.2021.790481)

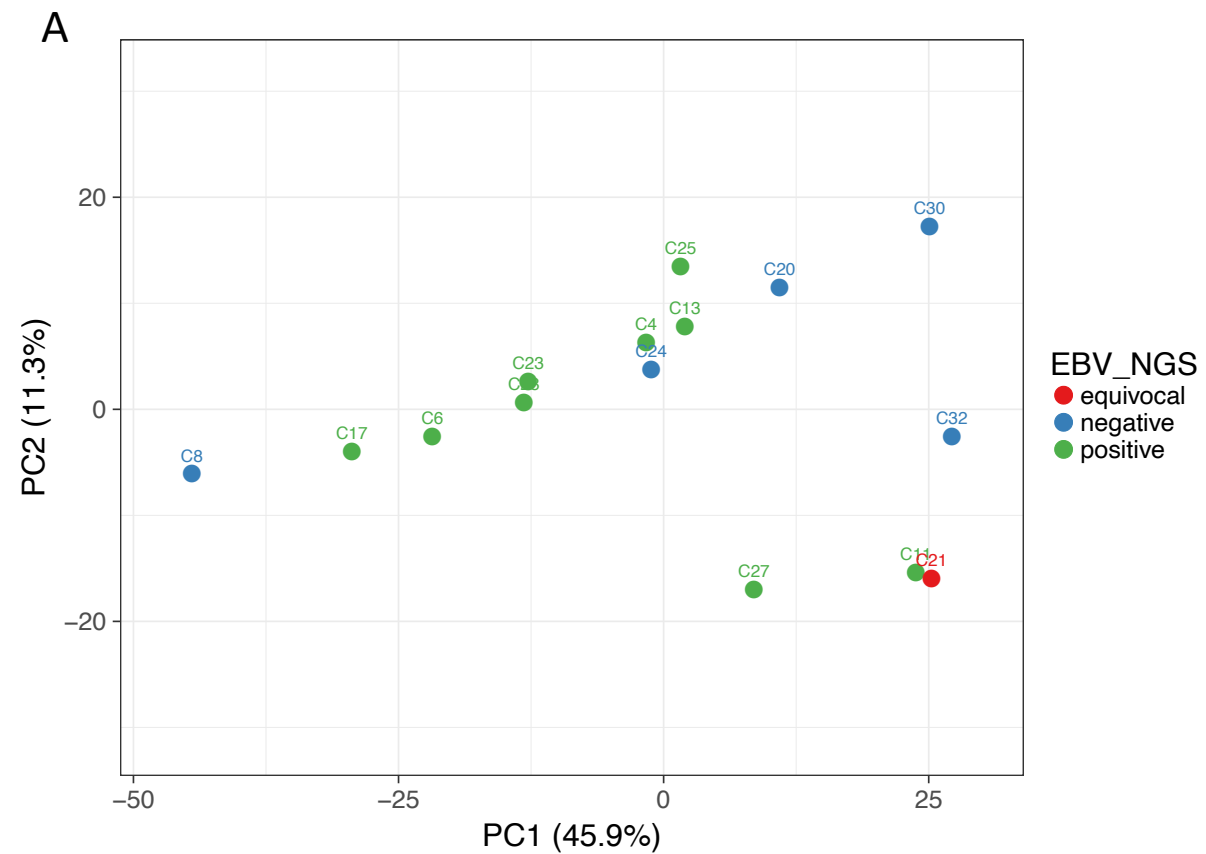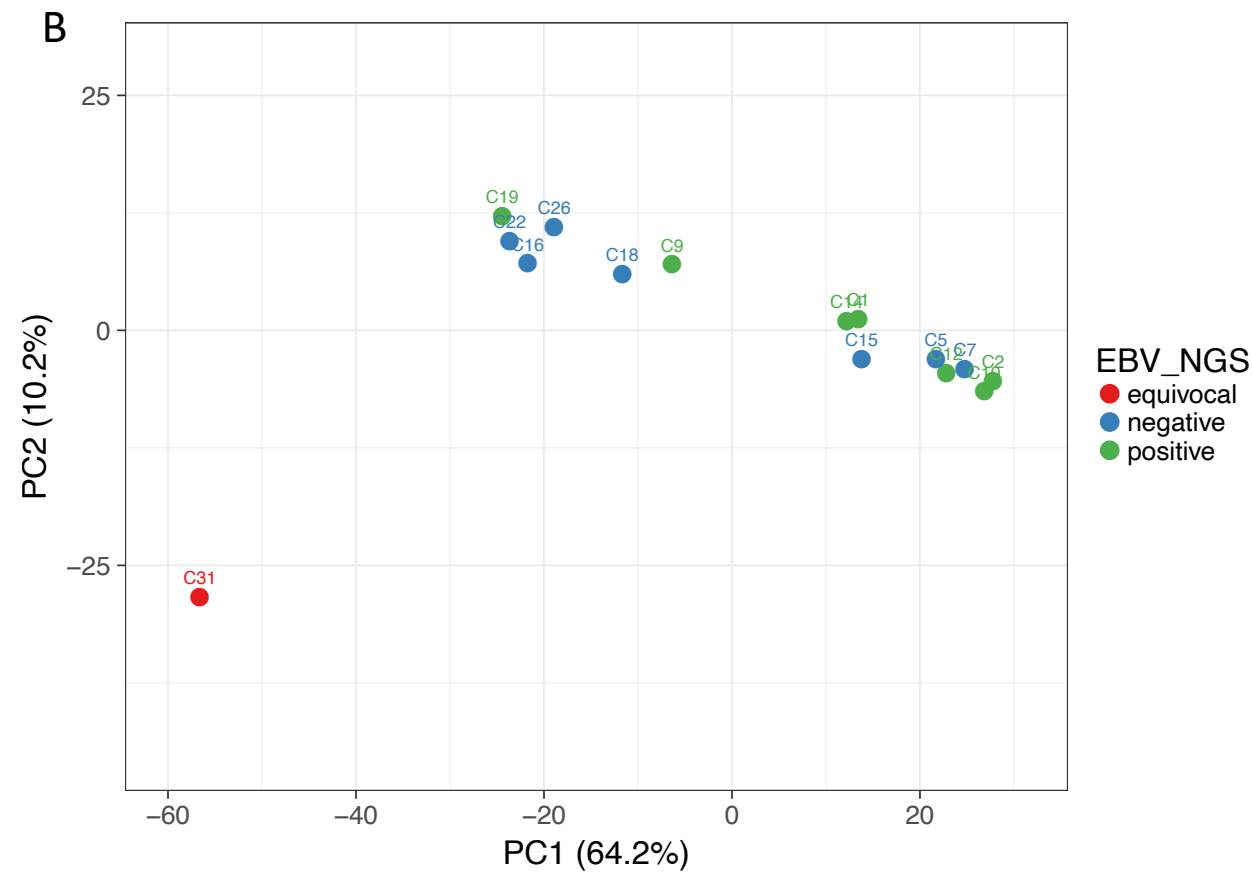

Supplement: Supplementary Figure 1 — PCA plot of the transcriptional profile of (A) EBV positive and EBV negative monomorphic, polymorphic, and classic Hodgkin (mpc)-PTLD cases and (B) EBV positive and EBV negative FFH-PTLD samples. EBV status was determined by NGS. [file DataSheet_1.pdf]
